# From Adipose Dysfunction to Multi-Organ Steatosis: Defining the Metabolic Steatotic Axis

**DOI:** 10.3390/cimb48020178

**Published:** 2026-02-04

**Authors:** Almir Fajkić, Yun Wah Lam, Rijad Jahić, Ivan Ćavar, Antonio Markotić, Andrej Belančić

**Affiliations:** 1 Department of Pathophysiology, Faculty of Medicine, University of Sarajevo, 71000 Sarajevo, Bosnia and Herzegovina; 2 Department of Health Sciences, School of Nursing and Health Sciences, Hong Kong Metropolitan University, Hong Kong SAR, China; wywlam@hkmu.edu.hk; 3 Department of Cardiology, University Clinical Center Sarajevo, 71000 Sarajevo, Bosnia and Herzegovina; rijadjahic2005@gmail.com; 4 Department of Immunology, School of Medicine, University of Mostar, 88000 Mostar, Bosnia and Herzegovina; ivan.cavar@mef.sum.ba; 5 Department of Physiology, School of Medicine, University of Mostar, 88000 Mostar, Bosnia and Herzegovina; antonio.markotic@mef.sum.ba; 6 Department of Basic and Clinical Pharmacology and Toxicology, Faculty of Medicine, University of Rijeka, 51000 Rijeka, Croatia

**Keywords:** Metabolic Steatotic Axis, multi-organ steatosis, axis-based staging

## Abstract

Steatosis extends beyond the liver to the pancreas, heart, and skeletal muscle, yet prevailing definitions remain narrowly organ-focused. This narrative review introduces the Metabolic Steatotic Axis (MSA) as a framework that captures the dynamic, bidirectional interactions among these organs, driving systemic metabolic dysfunction. We synthesize evidence linking lipotoxicity, inflammatory signaling, and endocrine cross-talk into a self-amplifying network accelerating insulin resistance, β-cell failure, and cardiometabolic risk. The MSA concept provides a rationale for axis-based staging systems and composite biomarker panels to quantify cumulative disease burden better and refine risk stratification. We highlight phenotypic heterogeneity within MSA stages, the possible hierarchy of organ vulnerability, and the implications for prognosis and therapy. Viewing pharmacological and lifestyle interventions through the MSA lens reframes them as systemic modulators rather than organ-specific treatments, underscoring the need for multi-organ endpoints in clinical trials. Finally, we outline priorities for longitudinal imaging, multi-omics integration, and global harmonization to translate the MSA from a conceptual construct to a clinically actionable paradigm. By unifying fragmented observations into a systemic model, the MSA has the potential to reshape disease classification, therapeutic strategies, and precision medicine in metabolic disorders.

## 1. Introduction

Steatosis is not a benign accumulation of fat but a clinically relevant process that drives type 2 diabetes, cardiovascular disease, and multi-organ dysfunction [1]. For decades, research and classification remained largely liver-centric. The transition from non-alcoholic fatty liver disease (NAFLD) to metabolic dysfunction-associated steatotic liver disease (MASLD) acknowledges metabolic rather than alcohol-related origins [2], while pancreatic steatosis (MASPD) further links ectopic fat to beta-cell failure and cardiometabolic risk [3].

Yet growing evidence shows that hepatic, pancreatic, cardiac, and skeletal muscle steatosis are interconnected through shared metabolic, endocrine, and inflammatory pathways [4,5]. Each organ contributes actively to systemic dysfunction rather than serving as a passive lipid depot; however, current classifications remain fragmented, lacking a framework to summarize multi-organ involvement and connect it to clinical risk.

To address this gap, we present the Metabolic Steatotic Axis (MSA) as a hypothesis-generating, working model. We recognize that the biology underneath is network-like and use “axis” here to describe a discernible backbone that relays, predominantly, the routes of lipid flux and endocrine–inflammatory signals across this set of relatively densely interconnected core organs (with liver as the hub and pancreas, heart, and skeletal muscle as downstream nodes with feedback loops). This presents a contrast to expansive “multi-organ crosstalk” or “lipotoxic network” concepts simply by formally emphasizing measurability and falsifiability. The MSA aims to link inter-organ interactions with uniformly measurable multi-organ readouts and candidate node-specific stress markers, in order to make testable statements on the hierarchy of organ vulnerability under lipid overload and for testing interventions, utilizing, as endpoints, not single-organ surrogates but bona fide multi-organ ones. Against this backdrop, the present narrative review integrates available evidence including human and preclinical data) and differentiates it in terms of general principles from direct organ-based evidence to convey the essential mechanistic and translational components that are needed to determine whether axis-oriented concepts could contribute to an optimized risk assessment and treatment decision beyond a focus only on specific organs.

## 2. Methods–Evidence Selection

This article is structured as a narrative review with elements of a scoping approach. We performed a targeted literature search in PubMed and Scopus up to August 2025 using combinations of the following terms: steatosis, multi-organ steatosis, adipose tissue dysfunction, hepatokines, myokines, cardiac steatosis, pancreatic steatosis, and metabolic dysfunction. Both human and preclinical studies were considered, but priority was given to prospective imaging studies, biomarker analyses, and interventional trials in humans. Reference lists of key articles and recent systematic reviews were screened to ensure comprehensive coverage. Because the concept of the MSA is novel and hypothesis-generating, no formal PRISMA methodology was applied; instead, the aim was to integrate available evidence to inform future research directions and clinical translation.

## 3. Initiation of Steatosis

Steatosis arises when the balance between lipid storage, oxidation, and export is disrupted. Under normal conditions, subcutaneous adipose tissue functions as a metabolic sink, storing excess triglycerides and protecting non-adipose organs from lipid overload. When this capacity is exceeded—through chronic caloric excess, adipose tissue inflammation and fibrosis, impaired mitochondrial oxidation, or defects in lipoprotein export—lipid intermediates spill into the circulation and infiltrate organs not designed for long-term storage [6,7]. In persistent energy surplus, adipocytes reach hypertrophic limits or become fibrotic, preventing further expansion. Inflamed adipose tissue is metabolically inflexible, accelerating lipolysis and releasing non-esterified fatty acids (NEFAs).

Obesity-induced adipose tissue inflammation is characterized by a shift in the adipokine profile toward a pro-inflammatory state, with decreased adiponectin and increased leptin, resistin, tumor necrosis factor alpha (TNF-alpha), and IL-6. This milieu activates canonical inflammatory pathways, notably nuclear factor kappa B (NF-kappaB) and c-Jun N-terminal kinase (JNK), which propagate metabolic inflammation and insulin resistance [8]. The activation of these pathways is further amplified by the NLR family pyrin domain containing 3 (NLRP3) inflammasome, which promotes maturation and secretion of IL-1beta and IL-18, contributing to systemic inflammation and metabolic dysfunction [9]. Toll-like receptor 4 (TLR4) signaling in adipose tissue macrophages further sustains this response, reacting to saturated fatty acids and lipopolysaccharide and promoting polarization toward a pro-inflammatory M1 phenotype [8].

Human studies and experimental models consistently demonstrate that these processes are interlinked; adipocyte hypertrophy and death in obesity increase macrophage recruitment and activation, amplifying pro-inflammatory cytokine release and worsening local and systemic inflammation [8]. The transition from anti-inflammatory (M2) to pro-inflammatory (M1) macrophage phenotypes is a hallmark of adipose tissue inflammation and is regulated by adipokines such as leptin and resistin, as well as local cytokine concentrations [10]. Notably, changes in adipokine and cytokine production can precede overt immune cell infiltration in early metabolic dysfunction [11].

Concurrently, systemic insulin resistance is reinforced through JNK and NF- kappaB signaling. When oxidative capacity becomes limiting, surplus fatty acids are redirected toward triglyceride synthesis and ectopic deposition [12]. In the liver, impaired very-low-density lipoprotein (VLDL) export further traps triglycerides within hepatocytes [13,14]. Collectively, these changes channel toxic intermediates, such as diacylglycerols and ceramides, into the liver, pancreas, heart, and skeletal muscle, where they disrupt signaling and initiate lipotoxic cascades.

At the cellular level, triglyceride accumulation is not intrinsically toxic; toxicity emerges when lipid handling loses temporal control. Lipid droplets act as dynamic buffers that sequester fatty acids away from signaling pools, but this protection depends on regulated droplet turnover and remodeling. When droplet dynamics become impaired, fatty acids are diverted into bioactive intermediates such as diacylglycerols and ceramides, amplifying insulin resistance and organ stress [15]. Lipophagy provides a key route for the controlled mobilization of droplet triglycerides, coupling lipid release to energetic demand and mitochondrial capacity; in obesity and metabolic dysfunction, autophagy programs can become blunted or maladaptive, leading to droplet persistence and prolonged exposure to lipotoxic intermediates [16]. Mitochondrial fatty acid oxidation represents a shared bottleneck across organs; once oxidative capacity is exceeded, beta-oxidation becomes inefficient, reactive oxygen species (ROS) generation increases, and organ-specific failure modes emerge. Triglyceride trapping is favored in the liver by oxidative stress and limited export capacity; contractile energetics are impaired in the heart by metabolic rigidity and lipid overload; insulin pathways are interfered with in skeletal muscle by lipid-derived signaling; and beta-cell dysfunction is accelerated in the pancreas by combined lipid and endoplasmic reticulum (ER) stress. These intracellular disruptions are not isolated; they generate circulating lipid species and stress signals that strengthen endocrine–inflammatory cross-talk and transform organ-local lipid handling failure into systemic metabolic stress and ectopic steatosis [17].

Current evidence from human imaging, biomarker studies, and experimental models supports the mechanistic link between lipid spillover, inflammatory signaling, metabolic inflexibility, and ectopic fat deposition the liver, pancreas, heart, and skeletal muscle. Chronic caloric excess, particularly Western-style diets, drives adipose tissue dysfunction and an inability to safely store surplus energy, leading to spillover of NEFAs and triglycerides into non-adipose tissues. This ectopic lipid accumulation is a central driver of insulin resistance and metabolic syndrome, shown in human and animal models of lipodystrophy and overnutrition, where subcutaneous adipose tissue cannot buffer excess energy, and pathological fat accumulates in liver, muscle, and pancreas [18,19]. The inability to confine lipids to adipose tissue marks a threshold beyond which the risks of diabetes, cardiovascular disease, and multi-organ failure increase sharply. Steatosis should therefore be recognized as a systemic phenomenon and a harbinger of systemic decline.

## 4. Organs of the Metabolic Steatotic Axis

**Figure 1:**
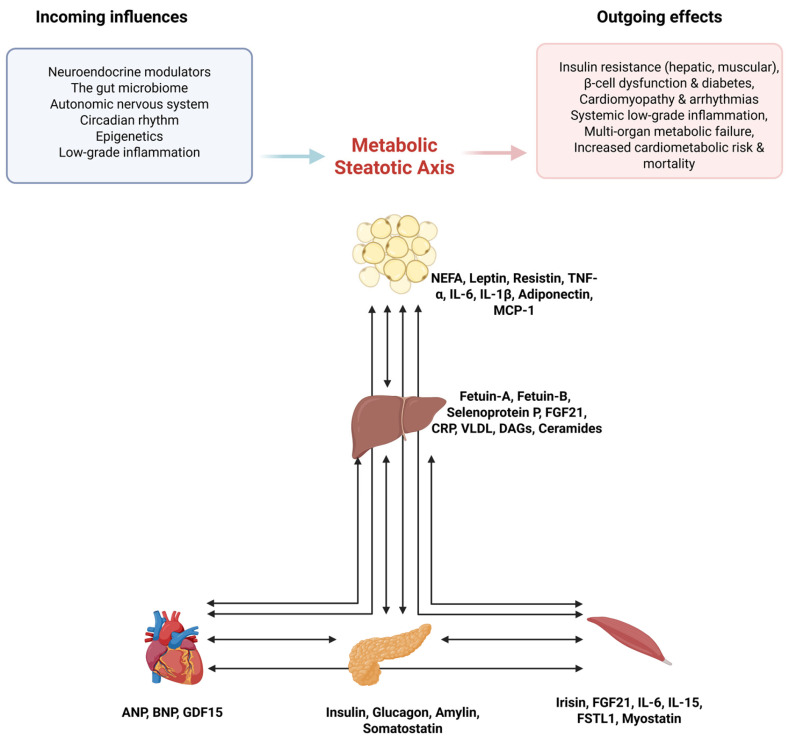
Conceptual model of the Metabolic Steatotic Axis: organ interactions, influencing factors, and systemic outcomes. Detailed evidence for each inter-organ interaction, including classification as either human or preclinical studies, and key references, is provided in Table 1. Created in BioRender. Fajkić, A. (2025) https://BioRender.com/q8bh5i9.

While animal models and mechanistic studies support the concept of ectopic lipid accumulation in multiple organs, direct evidence from human studies is robust for the brain (specifically the hypothalamus) and blood vessels, but remains limited or indirect for the gonads and lungs [20,21,22]. Given the scarcity of mechanistic and longitudinal data, these sites are not included in the core definition of the MSA but warrant further investigation as potential modifiers of systemic metabolic dysfunction. However, the liver, pancreas, heart, and skeletal muscle are selected to define the MSA because each of these four organs is both a major site of lipid deposition and an active source of signals that propagate dysfunction, making them uniquely positioned to define the axis (Figure 1).

### 4.1. Liver

The liver, as the central metabolic hub, is the first organ exposed to this lipid overflow. Hepatocytes accumulate triglycerides and toxic lipid intermediates such as diacyl-glycerol (DAG) and ceramides. These molecules activate protein kinase C epsilon (PKCε) and interfere with insulin receptor substrate signaling, while mitochondrial overload leads to excessive production of ROS. Importantly, the steatotic liver does not remain a passive target. It secretes hepatokines, including fetuin-A, fetuin-B, fibroblast growth factor 21 (FGF21), and selenoprotein P, which modulate insulin sensitivity in muscle, alter β-cell function in the pancreas, and contribute to cardiovascular risk. The recognition that the liver actively contributes to systemic metabolic dysfunction forms the rationale for renaming NAFLD to MASLD [23].

The strongest evidence for liver fat as a central hub that monitors insulin resistance, atherogenic dyslipidemia, and multi-organ ectopic fat clustering across cohorts comes from longitudinal observations showing that changes in hepatic fat correspond with changes in systemic metabolic risk [24]. Preclinical research relating hepatic lipid handling failure (overflow, VLDL-triglycerides export limits, hepatokines, inflammatory activation) to downstream organ dysfunction primarily supports mechanistic plausibility [25,26]. The temporal order (whether adipose dysfunction and spillover precede hepatic steatosis in all phenotypes) and the possibility that lipid turnover state (droplet dynamics, oxidative capacity, inflammatory context) is more informative than total hepatic fat remain important uncertainties.

### 4.2. Pancreas

The pancreas represents the second node of the axis. Emerging evidence suggests that exposure to lipids and hepatokines imposes severe stress on β-cells. Ceramides and DAG disrupt insulin signaling, while ER stress pathways, including protein kinase RNA-like endoplasmic reticulum kinase/eukaryotic translation initiation factor 2 alpha (PERK/eIF2α) and inositol-requiring enzyme 1 alpha/X-box binding protein 1 (IRE1/XBP1), are activated, leading to β-cell apoptosis and loss of secretory capacity. Amylin and other β-cell products further influence hepatic glucose output and vascular tone, extending the systemic consequences of pancreatic steatosis [27,28]. For these reasons, the emerging entity termed MASPD has been proposed as the pancreatic analog of MASLD, reflecting the fact that the pancreas is not a passive bystander but an active contributor to systemic failure.

Pancreatic fat is linked to beta-cell dysfunction and impaired glucose tolerance in humans, but the strength and consistency of this association vary greatly between cohorts, making a strong causal interpretation difficult [29,30]. More direct preclinical evidence connects lipid overload to oxidative stress, beta-cell ER stress, and reduced insulin secretory capacity. The two main areas of uncertainty are methodological (non-uniform quantification, fat compartmentalization) and causal directionality (i.e., whether pancreatic steatosis primarily reflects systemic ectopic fat burden or contributes independently to beta-cell failure) [31]. The absence of consistent measurement and longitudinal data required to distinguish between marker status and mechanistic contribution is a significant gap.

### 4.3. Heart

In the heart, lipid uptake through cluster of differentiation 36 (CD36) exceeds oxidative capacity. Mitochondrial overload generates ROS, while DAG and ceramides activate PKCε, impair calcium handling, and lead to contractile dysfunction, a hallmark of lipotoxic cardiomyopathy [32]. Human data support correlations between cardiac ectopic fat (myocardial and epicardial compartments) and functional phenotypes like diastolic impairment and cardiometabolic risk. Additionally, cardiac fat often clusters with pancreatic and hepatic steatosis [33,34].

The majority of the mechanistic understanding is preclinical and focuses on oxidative stress, metabolic inflexibility, mitochondrial lipid overload, impaired energetics, and possible paracrine effects of epicardial fat. The most pathogenic lipid compartment, whether dysfunction is caused by lipid quantity or lipid quality (intermediates), and the degree to which systemic insulin resistance and hemodynamic load confuse directionality are among the main uncertainties [35]. A compartment-resolved, phenotype-aware model that can distinguish between cardiac steatosis as a driver and a bystander of axis deterioration is the main gap.

### 4.4. Skeletal Muscle

Skeletal muscle, on the other hand, accumulates intramyocellular lipids that activate PKCθ, phosphorylate insulin receptor substrate 1 (IRS-1) on serine residues, and impair insulin signaling. This reduces glucose transporter type 4 (GLUT4) translocation and glucose uptake, establishing muscle insulin resistance [36]. Skeletal muscle lipid handling significantly influences axis-level glycemic and metabolic control in humans because it is closely associated with whole-body insulin sensitivity [37].

Myokines offer a plausible layer of inter-organ communication, while preclinical research supports a causal role for inflammatory signaling, mitochondrial stress, and lipid-derived intermediates (DAG, ceramides) in disrupting insulin pathways. The “athlete’s paradox,” which emphasizes context dependence and suggests that lipid localization, droplet turnover, and oxidative capacity may be more important than total intramyocellular triglyceride, highlights the central conflict [38,39]. Determining the precise cellular and physiological context in which muscle lipid storage stays adaptive versus when it transitions to lipotoxic signaling that spreads multi-organ dysfunction is the main area of uncertainty.

The links along the MSA are not unidirectional but bidirectional (Table 1). Across the axis, toxic lipid intermediates (diacylglycerols, ceramides), mitochondrial overload, and stress signaling (e.g., ER stress in β-cells, impaired calcium handling in cardiomyocytes, and PKC-mediated insulin-signaling defects in muscle) recur as common mechanisms of dysfunction. The steatotic liver does not simply accumulate fat but feeds back to adipose tissue through hepatokines such as fetuin-A, fetuin-B, and selenoprotein P, which worsen lipolysis and inflammation; in turn, excess fatty acids released from adipose tissue perpetuate MSA [40,41,42]. Pancreatic steatosis impairs insulin and glucagon secretion, reshaping adipose and hepatic metabolism, while at the same time being exacerbated by hepatic insulin resistance and systemic lipotoxicity. The heart responds to lipid overload by secreting natriuretic peptides that enhance adipose lipolysis [43]. Skeletal muscle releases myokines such as irisin and FGF21 that modulate adipocyte and hepatic function, but its own insulin resistance is aggravated by circulating hepatokines and adipokines [44]. These reciprocal interactions reinforce the constant dialogue between adipose tissue and downstream organs, establishing a reciprocal feedback mechanism in which no organ is merely a recipient of lipid excess; each contributes actively to systemic dysfunction [45].

Prospective MRI-proton density fat fraction (MRI-PDFF) studies in humans confirm that multi-organ steatosis predicts insulin resistance and cardiovascular risk, supporting findings from preclinical models [46,47].

**Table 1:** Inter-organ communication within the MSA.

Source Organ	Key Mediators	Target Organ	Main Effects in MSA Context	Evidence Level	Clinical Measurability	Ref
Adipose Tissue	NEFA, TNF-α, IL-6, Leptin, Resistin, ↓ Adiponectin, MCP-1	Liver	Hepatic steatosis, insulin resistance, inflammation	Human evidence	Serum NEFA, IL-6, adiponectin; MRI-PDFF	[48,49]
		Pancreas	β-cell lipotoxicity, impaired insulin secretion	Human + animal evidence	Serum NEFA, adipokines	[50,51,52,53,54]
		Heart	Lipotoxic cardiomyopathy, myocardial inflammation	Human evidence	MRI-PDFF, EAT thickness, circulating adipokines	[55,56,57]
		Skeletal Muscle	Muscle insulin resistance, mitochondrial dysfunction	Human evidence	Serum NEFA, MRI muscle fat	[58,59,60]
Liver	Fetuin-A, Fetuin-B, Selenoprotein P, FGF21, CRP, VLDL, DAGs, Ceramides	Adipose Tissue	↑ Lipolysis, systemic insulin resistance, inflammation	Human evidence	Serum Fetuin-A, FGF21, CRP (C- reactive protein)	[61,62,63]
		Pancreas	β-cell dysfunction, glucotoxicity, impaired insulin secretion	Preclinical only: pancreas models	Histology (β-cell mass, apoptosis), GSIS, oxidative/ER stress markers	[64,65,66]
		Heart	Endothelial dysfunction, cardiometabolic risk	Human evidence	CRP, Fetuin-A serum, MRI cardiac fat	[67,68,69]
		Skeletal Muscle	Muscle insulin resistance, impaired glucose uptake	Human evidence	Serum CRP, MRI muscle fat	[70,71,72]
Pancreas	Insulin, Amylin, Glucagon	Liver	↑ Lipogenesis, altered glucose metabolism	Human evidence	Serum insulin, glucagon	[73,74,75]
		Adipose Tissue	↑ Lipogenesis, adipose inflammation	Human evidence	Circulating amylin	[76,77,78]
		Heart	Myocardial stress, hypertrophy	Preclinical only: cardiac models	Histology (fibrosis, apoptosis), echocardiography (LVEF, FS), hemodynamic studies (LVEDP, dp/dt)	[79,80,81,82]
		Skeletal Muscle	Muscle insulin resistance, metabolic inflexibility	Human evidence	Serum insulin, glucose uptake studies	[83,84]
Heart	Natriuretic peptides (ANP, BNP)	Adipose Tissue	↑ Lipolysis, altered adipocyte metabolism	Human evidence	Plasma BNP, ANP	[85,86,87]
		Liver	Modulation of hepatic fat metabolism, lipid turnover	Preclinical only: liver models	Histology (steatosis, fibrosis), hepatic oxidative stress, lipid metabolism genes)	[88,89]
		Pancreas	Indirect effects via altered glucose and lipid handling	Human + animal evidence	Circulating natriuretic peptides	[90,91]
		Skeletal Muscle	Improved insulin sensitivity, metabolic flexibility	Human evidence	Muscle glucose uptake studies	[92,93,94]
Skeletal Muscle	Myokines (Irisin, IL-6, FGF21)	Liver	↑ FA oxidation, ↓ steatosis, improved glucose metabolism	Human evidence	Serum IL-6, FGF21, Irisin	[95]
		Adipose Tissue	Browning of white adipose tissue, ↑ energy expenditure	Animal evidence	Circulating myokines	[96,97]
		Pancreas	Modulation of insulin secretion and β-cell stress	Preclinical only: pancreas models	Histology (β-cell mass, apoptosis), GSIS, oxidative/ER stress markers	[98,99,100,101]
		Heart	Cardioprotective effects, improved myocardial metabolism	Human + animal evidence	Circulating FGF21, Irisin	[102,103,104]

↑ increase, ↓ decrease.

Inflammation acts as the systemic glue of the axis. Lipids and endotoxins activate TLR4 signaling in hepatocytes, adipocytes, and immune cells, resulting in the activation of NF-κB and JNK. Simultaneously, ceramides and ROS activate the NLRP3 inflammasome, leading to the release of IL-1β and IL-18. These cytokines circulate between organs, perpetuating tissue injury and sustaining low-grade inflammation. In this way, inflammatory signaling transforms localized steatosis into a self-reinforcing, multi-organ process [105]. The gut microbiome also contributes by increasing intestinal permeability and endotoxin release, activating TLR4 signaling in the liver and adipose tissue. Short-chain fatty acids and microbial metabolites influence hepatic gluconeogenesis and pancreatic insulin secretion, linking the gut–liver–pancreas triad [106]. Immune cells reinforce the process; adipose tissue macrophages polarize toward a pro-inflammatory M1 phenotype, while hepatic Kupffer cells amplify local and systemic cytokine release [107].

## 5. Modulators of the Metabolic Steatotic Axis (MSA)

### 5.1. Neuroendocrine Modulators of the MSA

The neuroendocrine layer is the missing architecture without which the MSA remains anatomically described but mechanistically incomplete. The liver, pancreas, heart, and skeletal muscle form its visible plane, yet the rhythm that keeps them in tune originates higher, within the hypothalamus, pituitary, and adrenal–thyroid complex. These centers decode nutritional, hormonal, and inflammatory cues and synchronize metabolic activity across organs. When this dialogue falters under chronic caloric load or inflammatory stress, communication noise becomes metabolic distortion.

#### 5.1.1. The Hypothalamus: Central Lipotoxicity as the First Signal

The hypothalamus acts as the brain’s metabolic compass. Within its arcuate (ARC), paraventricular (PVN), and lateral (LHA) nuclei, opposing neuronal sets, pro-opiomelanocortin (POMC) and neuropeptide Y/agouti-related peptide (NPY/AgRP), continuously negotiate hunger, satiety, and energy expenditure [108]. Under chronic nutrient excess, lipids infiltrate neurons and glia, generating “hypothalamic lipotoxicity” [109]. The result is ER stress, NF-kB activation, and glial inflammation that blunt leptin and insulin signaling, producing central lipid resistance, an early inflection point of systemic obesity and metabolic syndrome [110].

Experimental models confirm that hypothalamic lipid accumulation precedes hepatic steatosis and insulin resistance, with rapid rises in ceramides and diacylglycerols before histological fat appears in the liver [111,112]. As hypothalamic insulin resistance develops, sympathetic tone escapes inhibition, driving adipose lipolysis and elevating circulating FFAs that seed hepatic lipid overload [113]. The hypothalamus thus functions as an upstream ignition point of the MSA.

#### 5.1.2. Pituitary Relay: Endocrine Amplification

The pituitary converts hypothalamic signals into systemic endocrine commands via the hypothalamic-pituitary-adrenal (HPA), hypothalamic-pituitary-thyroid (HPT), and hypothalamic-pituitary-gonadal (HPG) axes, extending central rhythm into peripheral metabolism.

Chronic HPA activation and cortisol excess, as in Cushing’s syndrome, promote visceral adiposity, insulin resistance, and ectopic lipid storage [114]. Glucocorticoids enhance hepatic lipogenesis and proteolysis–lipolysis, sustaining neuroendocrine lipotoxicity [115]. Thyroid hormones (T3, T4) regulate mitochondrial biogenesis and beta-oxidation; even mild hypothyroidism or receptor resistance predisposes one to MASLD and cardiac steatosis [116]. TRbeta-selective thyromimetics (e.g., resmetirom) support the finding that restoring thyroid signaling can reduce hepatic fat without TRalpha-dominant toxicity [117].

Sex steroids complete the circuit. Estrogen deficiency shifts fat centrally and in-creases NAFLD risk, while low testosterone in men and androgen excess in women favor visceral adiposity and insulin resistance [118]. Through ER-alpha/beta signaling, estrogens preserve lipid partitioning and insulin sensitivity; their loss removes an anti-inflammatory brake on the MSA [119]

#### 5.1.3. Adrenal and Autonomic Crosstalk

The adrenal medulla and sympathetic nervous system provide the axis’ rapid-response circuitry. Catecholamines mobilize lipolysis, flooding the circulation with NEFAs that infiltrate the liver and pancreas, accelerating steatosis and insulin resistance [120]. Chronic sympathetic overdrive impairs GLUT4 translocation in muscle, stimulates hepatic gluconeogenesis, and suppresses insulin release—especially when parasympathetic tone is low [121]. Therapeutic dampening of sympathetic output, whether behavioral or pharmacologic, consistently improves metabolic indices, underscoring the role of autonomic imbalance in sustaining the MSA [122].

Viewed together, these systems form a neuroendocrine–metabolic continuum where lipid toxicity, inflammation, and hormonal drift reinforce one another. The hypothalamus–pituitary–adrenal–thyroid network not only regulates but also mirrors the state of peripheral organs, closing the feedback loop between central command and metabolic outcome. Multi-organ steatosis thus emerges not as a peripheral accident of obesity but as the visible consequence of a central loss of metabolic coherence.

### 5.2. Extended Regulatory Modulators of the MSA

Beyond classical neuroendocrine organs, several interrelated systems exert regulatory control over the MSA, influencing lipid partitioning, insulin sensitivity, and inter-organ communication. These include the gut–brain–liver axis, autonomic nervous system, circadian and sleep–wake regulators, and epigenetic and inflammatory signaling hubs. Their integration into the MSA model provides a system-level understanding of how environmental, behavioral, and molecular inputs converge on multi-organ steatosis.

#### 5.2.1. The Gut–Microbiome Interface: A Peripheral Neuroendocrine Organ

The gastrointestinal tract and its microbiota function as an essential metabolic interface, acting as both nutrient sensor and endocrine organ. Enteroendocrine cells release glucagon-like peptide-1 (GLP-1), glucose-dependent insulinotropic polypeptide (GIP), peptide YY (PYY), and ghrelin in response to luminal cues, modulating appetite, insulin secretion, and energy balance; GLP-1 and PYY promote satiety and glycemic control, GIP supports glucose-dependent insulin release, and ghrelin rises during fasting to stimulate hunger [123].

The microbiota shapes these hormonal circuits through metabolites such as SCFAs, which stimulate GLP-1 and PYY secretion and improve glycemic regulation [124]. With reduced microbial diversity, intestinal permeability increases and LPS enters the portal circulation, driving metabolic endotoxemia [125]. Subsequent activation of TLR4 and NF-kappaB signaling in hepatocytes and adipocytes sustains low-grade inflammation, insulin resistance, and hepatic steatosis [126].

Microbial metabolites also act as biochemical messengers linking gut and systemic metabolism. SCFAs, secondary bile acids, and tryptophan-derived indoles engage G protein-coupled receptors (GPCRs: GPR41, GPR43, GPR119) and nuclear receptors-peroxisome proliferator-activated receptor (PPARs: PPARalpha, PPARgamma, aryl hydrocarbon receptor), influencing hepatic gluconeogenesis, pancreatic beta-cell function, and hypothalamic inflammatory tone [44,46]. In adipose tissue, SCFAs promote browning and thermogenesis, extending their effects to energy expenditure [126].

Overall, the microbiome operates as a peripheral neuroendocrine organ, translating dietary patterns into hormonal and immunometabolic signals that converge on both central and peripheral nodes of the MSA.

#### 5.2.2. The Autonomic Nervous System and Vagal–Sympathetic Balance

The autonomic nervous system (ANS) forms a bidirectional bridge between the central nervous system and the peripheral organs of the MSA. Sympathetic overactivity drives adipose lipolysis, elevating plasma NEFAs that accumulate in the liver and pancreas and accelerate steatosis [120]. Adrenergic activation enhances fatty acid release, and sympathetic nerve activity correlates with hepatic lipid deposition in obesity and even fasting. Experimental blockade or hepatic denervation attenuates steatosis by reducing fatty acid uptake and modulating lipid trafficking, with effects largely independent of weight change or caloric intake [127]. Sympathetic signaling also sustains low-grade inflammation through cytokine activation [128].

Parasympathetic (vagal) tone exerts complementary, context-dependent effects. Cholinergic input to the liver is implicated in steatosis under obesogenic conditions, yet vagal disruption can prevent lipid accumulation and promote browning of white adipose tissue [129]. The vagus nerve facilitates insulin secretion and hepatic glucose uptake, linking autonomic balance to glycemic stability. Its anti-inflammatory arm, the cholinergic reflex, operates through alpha7-nicotinic receptors on macrophages, dampening tissue inflammation and supporting metabolic homeostasis [128].

In essence, the ANS mirrors the metabolic state it governs; sympathetic excess accelerates lipid overflow and inflammation, while vagal withdrawal removes a key brake, turning neural regulation into a metabolic amplifier within the MSA.

Experimental models indicate that intact vagal innervation is essential for lipid and mitochondrial homeostasis. Disruption of liver–brain circuitry, via vagotomy or ablation of parasympathetic cholinergic neurons, induces hepatic steatosis, reduces beta-oxidation, and impairs energy expenditure [129]. Loss of vagal input shifts hepatic metabolism toward glycolysis and lipogenesis, diminishes mitochondrial respiratory efficiency, and promotes hepatocellular lipid accumulation [130,131]. Conversely, vagal preservation or stimulation enhances mitochondrial respiration, increases energy expenditure, and mitigates steatosis in high-fat or high-carbohydrate feeding models [132]. These findings extend the MSA beyond biochemical regulation to include neural control, positioning dysautonomia as a mechanistic bridge between neuroendocrine imbalance and metabolic organ failure, and a potential entry point for bioelectrical modulation [132].

Dysautonomia in metabolic disease is marked by sympathetic hyperactivity and loss of parasympathetic coherence. In obesity, diabetes, and sleep disorders, this manifests as hepatic neuropathy and progressive metabolic disarray [133]. Vagal afferent and efferent fibers regulate hepatic lipid turnover, systemic energy balance, insulin sensitivity, and feeding- and reward-related behaviors [134,135]. The vagal network also safeguards mitochondrial integrity by sustaining biogenesis, mitophagy, and redox equilibrium [136]. When vagal tone declines and sympathetic drive dominates, this protective circuitry unravels, fusing neuroendocrine and metabolic dysfunction into a single pathological continuum [135,136].

#### 5.2.3. Circadian and Chronometabolic Regulators

Circadian rhythms act as temporal regulators of the MSA, aligning hormonal secretion, mitochondrial activity, and lipid turnover across organs. Core clock genes (BMAL1, CLOCK, PER, and CRY) maintain this synchrony, ensuring that metabolism oscillates predictably between storage and oxidation phases. When light–dark cycles are disrupted, as in shift work or nocturnal feeding, hepatic and hypothalamic clocks drift out of phase, desynchronizing cortisol, insulin, and melatonin release. The consequence is metabolic jet lag—nocturnal lipolysis, elevated NEFAs, and progressive hepatic fat accumulation—even in the absence of caloric overload [137,138,139].

This temporal misalignment intersects with inflammatory signaling. Cytokines such as IL-6, TNFα, and IL-1β exhibit circadian oscillations, and when these rhythms collapse, inflammation and metabolism reinforce one another. Microglial activation and peripheral macrophage polarization amplify this cross-talk, linking central inflammation to peripheral lipid dysregulation [140,141,142]. In this setting, melatonin and adiponectin, usually oscillating in opposition, lose their rhythmic coherence, a change that predicts multi-organ steatosis [143,144].

Restoring circadian order through timed feeding, structured light exposure, or chronotherapy may thus realign both the temporal and inflammatory dimensions of the MSA, transforming the rhythm from a passive backdrop into a therapeutic target.

#### 5.2.4. Epigenetic and Transcriptional Regulators

Epigenetic mechanisms form the molecular “memory” of the MSA, embedding metabolic experience into long-term gene regulation. DNA methylation and microRNA (miRNA) networks jointly define how lipid metabolism adapts—or maladapts—to nutritional and environmental stressors. Hypermethylation of the PGC-1α promoter, induced by high-fat, high-sugar exposure, suppresses mitochondrial biogenesis and β-oxidation, while enhancing SREBP-1c and PPARγ signaling—thereby shifting hepatic metabolism toward lipid storage and steatosis [145].

Circulating miRNAs act as post-transcriptional messengers linking adipose, hepatic, and endocrine signaling. Among them, miR-122-5p correlates with hepatic and adipose insulin resistance and elevated free fatty acids. At the same time, let-7d-5p and let-7f-5p inversely associate with lipolysis and inflammation, suggesting a fine-tuned regulatory circuit connecting lipid handling and immune tone [146].

Together, these epigenetic layers form the substrate of metabolic memory. In this phenomenon, early molecular reprogramming predisposes individuals to persistent metabolic dysfunction and multi-organ steatosis, even in the absence of ongoing insult.

#### 5.2.5. Inflammatory and Immune Neuroendocrine Integration

Chronic low-grade inflammation acts as both an effector and a regulator within the MSA. Cytokines such as IL-6, TNF-alpha, and IL-1beta signal across central and peripheral compartments; produced in the hypothalamus and peripheral tissues, they cross the blood–brain barrier or transmit via neural pathways to impair hypothalamic insulin sensitivity and disrupt neuroendocrine control of energy balance [147,148].

Within the hypothalamus, metabolic stress activates microglia through TLR4 with downstream NF-kappaB and JNK signaling, driving proinflammatory cytokine release and local gliosis [149]. Astrocytic NF-kappaB activation amplifies this response, further compromising central metabolic regulation [149]. This glial–endocrine–metabolic coupling links neuroinflammation to hepatic and pancreatic dysfunction [147].

Peripherally, adipose expansion recruits macrophages with a proinflammatory phenotype, sustaining cytokine release and impairing insulin signaling across organs [150]. This feed-forward circuit ensures hypothalamic activation not only mirrors but also drives systemic metabolic injury [150]. Neuroimmune circuits, including the vagus nerve–spleen–adipose axis, provide reciprocal feedback in which peripheral inflammatory cues reshape central neuroendocrine output, reinforcing the cycle of inflammation and metabolic disruption [151]. Together, this cytokine circuitry binds neural, endocrine, and immune layers of the MSA into a single pathophysiological continuum.

## 6. Clinical Implications of the Metabolic Steatotic Axis

We argue that viewing steatosis through the lens of the MSA may have direct implications for diagnosis. Current approaches remain heavily liver-centric, relying on ultrasound, transient elastography, or Magnetic Resonance Imaging–Proton Density Fat Fraction (MRI-PDFF) to quantify hepatic fat. While these tools are valuable, they fail to capture the true burden of disease when steatosis is present in multiple organs [107]. An axis-oriented diagnostic strategy would involve integrated imaging protocols capable of quantifying both hepatic and pancreatic steatosis, coupled with biomarkers that reflect cardiac and skeletal muscle lipid infiltration. Such an approach would provide a more comprehensive assessment of disease burden and risk.

The prognostic value of the axis is equally important. Patients with MASLD alone are at risk of progression to type 2 diabetes and cardiovascular disease, but those with combined MASLD and MASPD deteriorate more rapidly. Multi-organ steatosis amplifies insulin resistance, accelerates β-cell failure, and increases the likelihood of cardiovascular events [152]. Prognostic models that fail to consider the extent of axis involvement may underestimate true risk. Incorporating multi-organ steatosis into risk stratification frameworks would allow for earlier identification of high-risk patients and more precise allocation of preventive strategies.

Another translational implication of the MSA framework lies in phenotypic diversification. Not all patients with a similar extent of multi-organ involvement exhibit the same risk trajectories; some progress rapidly to cardiometabolic events while others remain stable for years. This heterogeneity likely reflects differences in organ sequence involvement, inflammatory tone, and metabolic reserve. Developing phenotypic clusters within each MSA stage—such as inflammatory-dominant versus lipid-dominant profiles—could allow for the tailoring of therapeutic intensity and follow-up intervals. Such phenotyping could support a shift from one-size-fits-all staging toward precision risk stratification aligned with the biological underpinnings of axis dysfunction.

An unresolved question is whether organs along the MSA follow a hierarchy of metabolic vulnerability driven by their intrinsic energy demands. The heart and skeletal muscle, with continuous ATP turnover, may reach mitochondrial thresholds earlier than the liver or pancreas under conditions of lipid overload [153,154]. Such energetic fragility could explain why myocardial steatosis is associated with early diastolic dysfunction even in patients without advanced hepatic disease. Conversely, the liver’s central role in lipid trafficking may render it a dominant amplifier once steatosis is established. Mapping this energetic hierarchy could refine our understanding of why clinical trajectories differ despite similar systemic risk profiles.

Viewing steatosis as an axis also has potential therapeutic implications. Agents such as GLP-1 receptor agonists, sodium-glucose cotransporter 2 (SGLT2) inhibitors, and thiazolidinediones demonstrate beneficial effects across several nodes of the axis, reducing hepatic fat, improving β-cell function, and lowering cardiovascular risk [155,156,157]. However, this requires confirmation in prospective studies designed explicitly with axis-wide endpoints. Lifestyle interventions, including caloric restriction and structured exercise, similarly exert systemic effects [158]. These therapies may be best regarded as modulators of the axis rather than treatments confined to a single organ. Future clinical trials should adopt multi-organ endpoints to measure true efficacy.

## 7. Operationalizing the Axis: Staging and Biomarkers

The development of robust biomarkers is necessary to detect and quantify axis activity, enabling future clinical translation. A proposed Axis Signature Panel (ASP) would combine markers from each node. For the liver, alanine aminotransferase (ALT) and cytokeratin-18 (CK-18) fragments reflect hepatocellular injury [159]. For the pancreas, circulating exosomal microRNAs and variability in pancreatic enzyme levels could provide early signals of steatosis [160]. For the heart, high-sensitivity troponin and fatty acid-binding protein 3 reflect metabolic stress and injury [161,162]. For skeletal muscle, creatine kinase, FGF21, and irisin could indicate lipid accumulation and impaired insulin sensitivity [163]. Longitudinal biomarker cohorts demonstrate that elevated IL-6 and CK-18 predict multi-organ lipid accumulation and adverse metabolic outcomes in humans [164,165]. While individually imperfect, the integration of these signals into a composite Axis Index (AXI) could offer a multidimensional readout of axis activity.

In addition to lipid and inflammatory mediators, one-carbon metabolism represents a crucial yet often overlooked regulatory layer linking micronutrient status to systemic metabolic dysfunction [166]. Deficiencies in vitamin B12 and folate impair methylation capacity and mitochondrial function, whereas choline deficiency disrupts VLDL assembly and hepatic lipid export, collectively exacerbating hepatic steatosis, insulin resistance, and cardiovascular risk. Incorporating vitamin B12, folate, and choline into the ASP may therefore enhance its ability to capture metabolic stress, improve risk stratification, and open avenues for nutritional and pharmacological interventions targeting the MSA.

Such a panel would require rigorous validation in prospective cohorts but could ultimately support the stratification of patients along the MSA continuum, guiding both therapeutic choices and the monitoring of intervention efficacy.

The broader implication is a shift in perspective; instead of assessing steatosis as isolated organ pathology, the MSA framework enables clinicians and researchers to view lipid accumulation as a systemic process with cumulative impact. As risk models based solely on hepatic fat underestimate patient vulnerability, this axis-based assessment could refine prognosis, identify high-risk phenotypes earlier, and drive personalized therapeutic strategies. Moreover, clinical trials adopting axis-oriented endpoints would capture the true efficacy of interventions beyond a single organ, facilitating future translation into clinically relevant outcomes.

By framing the MSA in terms of biomarkers and clinical endpoints, the concept moves beyond a descriptive hypothesis toward testable clinical constructs. Importantly, these proposals should be viewed as provocative starting points rather than finalized instruments, intended to stimulate systematic validation and integration into clinical research.

Building on the conceptual framework presented above, Table 2 summarizes a proposed minimal clinical panel, standardized imaging protocol, composite indices, and trial endpoints to enable systematic validation and clinical translation of the MSA framework.

## 8. Future Directions

The establishment of the MSA as a conceptual framework must be followed by rigorous efforts to test, validate, and operationalize it. A priority is the development of integrated imaging protocols that can simultaneously quantify steatosis in the liver and pancreas, with add-on techniques for cardiac and skeletal muscle. MRI-based approaches already provide accurate measures of hepatic and pancreatic fat, and their adaptation into standardized multi-organ protocols could provide the first clinical tools for axis assessment.

As part of this process, we propose that the four-level staging system (MSA-0 to MSA-3) should be regarded solely as a hypothesis-generating concept requiring prospective validation rather than a definitive clinical taxonomy. The model envisions single-organ steatosis, most often in the liver, as MSA-0; bi-organ involvement, commonly liver and pancreas, as MSA-1; tri-organ disease involving the liver, pancreas, and either the heart or skeletal muscle, as MSA-2; and full-axis involvement of all four organs as MSA-3. Framing this model within future research allows its clinical utility to be explored through imaging, biomarker development, and longitudinal cohort studies before any formal adoption.

If validated in prospective cohorts, such a framework could provide a scaffold to assess cumulative disease burden and enable research and clinical stratification. Whereas traditional classifications focus on within-organ severity, the MSA framework captures systemic impact across multiple sites. Using the number of affected organs offers a more reproducible criterion across settings and may reduce under-recognition; advanced axis involvement can remain clinically silent under current liver-centric paradigms. A patient at MSA-3 may show extensive multi-organ steatosis yet present with normal liver enzymes [171,172,173], thereby evading current screening strategies. Such scenarios highlight the risk of misclassification and the potential value of axis-based diagnosis and risk stratification.

Second, the axis should be characterized at the molecular level. Omics technologies, including lipidomics, metabolomics, and proteomics, could define circulating and tissue-specific “axis signatures,” clarify molecular coherence, and identify candidate biomarkers for clinical translation [174,175]. Such work could converge with the proposed Axis Signature Panel, advancing it from hypothesis generation toward validated assays.

Third, incorporating the neuroendocrine dimension underscores the need for axis-wide biomarkers capturing central and peripheral dysfunction, such as circulating cortisol, TSH/free T3 ratios, the leptin/adiponectin index, and markers of hypothalamic inflammation or sympathetic tone. Future iterations of the MSA model should explicitly recognize the hypothalamus and its hormonal axes as upstream regulatory hubs and potential therapeutic targets.

Finally, longitudinal studies are essential to map progression along the axis. Current evidence is largely cross-sectional, documenting associations rather than trajectories. Prospective cohorts with imaging, biomarkers, and outcomes tracked over time could clarify whether patients move from MSA-0 to MSA-3 and at what pace and identify “tipping points” where intervention is most effective.

The axis concept should also be embedded in therapeutic development and clinical trials. Drugs should no longer be evaluated solely for hepatic fat or glycemic control but for their ability to modulate the axis as a whole [176,177]. Lifestyle interventions, pharmacological agents, and emerging bioelectrical or metabolic therapies could be tested using multi-organ endpoints aligned with disease biology.

Reversibility of steatosis may not be uniform across organs. Hepatic and skeletal muscle fat often regress with weight loss or exercise, but beta-cells and cardiomyocytes show limited regenerative capacity, suggesting possible “points of no return” [178,179]. Another open question is whether a consistent “organ leader” drives systemic decompensation. In some patients, the liver appears dominant, while in others the pancreas or skeletal muscle may trigger failure. Clarifying these trajectories would refine staging and therapeutic timing.

Future research should progress beyond descriptive studies toward adaptive, multi-phase trials integrating multi-organ composite endpoints, real-time biomarker monitoring, and mechanistic substudies across MSA stages. AI-driven multi-omics platforms could fuse imaging, lipidomic, and proteomic data with longitudinal outcomes to identify molecular tipping points and high-risk subgroups. Federated learning across global consortia could harmonize imaging protocols, biomarker assays, and analytical frameworks, supporting globally validated staging systems and precision therapeutic algorithms.

Moving the MSA from a proposal to a clinically applicable framework will also require methodological and regulatory harmonization. Substantial heterogeneity across imaging protocols, biomarker panels, and grading systems limits cross-cohort comparability and hinders translational progress. Dedicated international consortia could enable standardized multi-organ imaging and integrated multi-omics platforms, while supporting consensus on staging criteria and risk thresholds analogous to fibrosis scoring systems in hepatology. Such harmonization would enhance reproducibility and facilitate the incorporation of axis-based endpoints into interventional trials and, if validated, future clinical guidance.

## 9. Conclusions

Steatosis is no longer adequately described by organ-centric definitions. Evidence consistently shows that the liver, pancreas, heart, and skeletal muscle are not passive repositories of lipid excess but active players in a coordinated process driven by metabolic dysfunction. Adipose tissue dysfunction initiates this cascade, while each affected organ amplifies it through endocrine, metabolic, and inflammatory signaling.

The MSA provides a unifying framework that integrates these insights. By conceptualizing steatosis as an axis, we move beyond fragmented terminology toward a systemic model that can better explain clinical outcomes, inform prognosis, and align therapies with their multi-organ effects.

The challenge for the field is clear: steatosis should be studied, classified, and treated as an axis disorder. Here, the “axis” reflects a network of reciprocal inter-organ relationships rather than a rigid linear construct, emphasizing the systemic nature of steatotic disease. An axis-based perspective may refine disease classification and re-shape screening practices, risk stratification, and therapeutic endpoints across metabolic medicine.

## Figures and Tables

**Table 2:** Proposed clinical pathway for applying the MSA in practice.

Minimal Clinical Panel (Baseline ± 6–12 Months)	Metabolic Core: HbA1c, Fasting Insulin (HOMA-IR), Triglycerides, HDL-C, ALT, AST, GGT Inflammatory Core: hs-CRP, IL-6 Endocrine/Lipotoxicity Markers: Adiponectin, Leptin (Leptin/Adiponectin Ratio), FGF21 Extended (Where Available): CK-18 (M30/M65), Fetuin-A/B, Selenoprotein P, NT-proBNP, FABP3/5, Irisin, NEFA, Ceramides/DAG
Imaging Core	Mandatory: Liver and pancreas MRI-PDFF (baseline, 6–12 months) Optional: Epicardial fat (echocardiography/MRI), myocardial fat fraction (MRI/MRS), skeletal muscle PDFF/MRS (m. vastus lateralis/gluteus) Thresholds: ≥30% relative reduction or ≥3 absolute PDFF points = clinically meaningful *
Composite Indices (Research Tools)	AXI-Core (0–100): Weighted composite of liver/pancreas PDFF, FGF21, leptin/adiponectin ratio, hs-CRP AXI-OrgCount (0–4): Number of organs above predefined PDFF/biomarker thresholds AXI-Inflam (0–3): Elevated hs-CRP (e.g., >2 mg/L or study-specific thresholds), IL-6 in upper quantiles, CK-18 above established NAFLD/NASH cut-offs **
Trial Endpoints (12–24 weeks)	Primary: Composite AXI-Core change OR hierarchical win-ratio across liver/pancreas PDFF, FGF21, leptin/adiponectin, hs-CRP Secondary: Proportion responders (≥30% PDFF or ≥20% AXI-Core reduction), intramyocellular fat (MRS), epicardial fat, HbA1c, HOMA-IR, NT-proBNP Exploratory: Lipidomics (ceramides/DAG), exosomal miRNAs, stratification by AXI-Inflam

* MRI-PDFF response thresholds (e.g., ≥30% relative reduction or ≥3 absolute PDFF points) are based on clinical evidence, including interventional studies and meta-analyses, demonstrating associations between changes in MRI-PDFF and histological improvement in the liver [46,47,167]. Thresholds for pancreatic and myocardial PDFF require further validation in dedicated longitudinal cohorts. ** Proposed inflammatory thresholds are derived from heterogeneous evidence across prospective cohorts, cross-sectional studies, and meta-analyses in NAFLD/MASLD and cardiometabolic populations. Elevated hs-CRP (>2 mg/L) has been linked to increased hepatic steatosis, fibrosis, and cardiometabolic risk in multiple cohorts [168,169,170]. IL-6 in upper quantiles has been associated with insulin resistance and hepatic steatosis [164], while CK-18 fragments remain the most validated noninvasive marker for hepatocyte apoptosis in MASH [165]. These cut-offs should be regarded as exploratory and require prospective validation for multi-organ steatosis.

## Data Availability

No new data were created or analyzed in this study.

## Abbreviations

The following abbreviations are used in this manuscript:

CD36	Scavenger Receptor Class B
eIF2α	Eukaryotic Initiation Factor 2 Alpha
FGF21	Fibroblast Growth Factor 21
IRE1	Inositol-Requiring Enzyme 1
JNK	c-Jun N-terminal Kinase
NF-κB	Nuclear Factor Kappa-light-chain
NLRP3	NOD-like Receptor Family Pyrin Domain-containing 3
PERK	PKR-like ER Kinase
PKC	Protein Kinase C
TNF-α	Tumor Necrosis Factor-alpha
TLR4	Toll-like Receptor 4
XBP1	X-box Binding Protein 1
